# Development and Validation of a Malay Version of the Questionnaire on Pornography Attitudes and Exposure for Youth in Kelantan

**DOI:** 10.21315/mjms2020.27.2.14

**Published:** 2020-04-30

**Authors:** Azriani Ab Rahman, Mohd Ismail Ibrahim, Razlina Abdul Rahman, Wan Nor Arifin, Mokhtarrudin Ahmad

**Affiliations:** 1 Department of Community Medicine, School of Medical Sciences, Universiti Sains Malaysia, Kelantan, Malaysia; 2 Department of Family Medicine, School of Medical Sciences, Universiti Sains Malaysia, Kelantan, Malaysia; 3 Unit of Biostatistics and Research Methodology, School of Medical Sciences, Universiti Sains Malaysia, Kelantan, Malaysia; 4 Faculty of Applied Communication, Multimedia University, Selangor, Malaysia

**Keywords:** pornography questionnaire, youth, development, validation

## Abstract

**Background:**

The aim of the study is to develop and validate the Malay version of the questionnaire assessing the extent of pornography exposure (PORQUE) among youth in Kelantan.

**Methods:**

‘Youth’ was defined as a person aged between 15 and 24 years, while ‘pornography’ refers to any material that depicts sexual activity or erotic behaviour. The development phase consisted of a literature review, an expert panel review, face validation and a pre-test. Fifty college students from a randomly selected higher learning institutions were invited to participate in the pilot test, whereas 150 and 198 different students from the same institutions participated in exploratory factor analysis (EFA) and confirmatory factor analysis (CFA), respectively.

**Results:**

EFA suggested a five-factor solution with factor loadings ranging from 0.449 to 0.891 and a Cronbach’s alpha ranging from 0.904 to 0.966. The CFA of the attitude questions also proved a good fitting model with good fit indices: comparative fit index (CFI) robust = 0.907; Tucker-Lewis fit index (TLI) robust = 0.901; root mean square error of approximation (RMSEA) robust = 0.073; standardised root mean square residual (SRMR) = 0.060. The factor loadings ranged from 0.544 to 0.906, whereas the Raykov’s rho ranged from 0.886 – 0.974.

**Conclusion:**

Based on EFA and CFA, the attitude sections of the newly developed Malay version of the PORQUE were found to be psychometrically valid.

## Introduction

Pornography has penetrated our society globally in the past 20 years through easily accessible technology, such as smart phones and the internet. Despite being labelled as a taboo or sensitive issue among people in Malaysian society, our youth has been exposed to pornography as early as 11 years old (1–2). Pornography can be defined as sexually explicit material (SEM) that includes genitals or sexual activities, such as masturbation, oral sex or unconcealed vaginal and anal penetration, intended to create sexual arousal in its consumers (3–5).

Worldwide, pornography exposure among youth ranges from 19% in a US study to 70% in a study in Australia (6–7). On the other hand, the prevalence of intentional exposure was found to be slightly lower, ranging from 7% in a US study to 59% in a study in Taiwan (8). In Malaysia, a population survey revealed an increasing trend of pornography exposure over a six-year period, with higher exposure with increasing age groups (9–10). Another survey by a Malaysian non-governmental organisation (NGO) exhibited 80% exposure to pornography, with intentional exposure in half of the respondents (11).

Despite the increasing negative evidence of pornography, studies specifically focusing on pornography are still lacking in Malaysia. There is no reliable measurement tool that can be used to assess pornography exposure, consumption patterns and attitudes on a whole, be it locally or internationally. Most previous studies on pornography have been observational in nature, providing descriptive results (6, 7, 12). Moreover, some instruments used in other studies have been deemed as not suitable in the Malaysian context due to different levels of acceptance of pornography among different cultures (13–14).

Hence, this study aimed to develop and validate a tool for the measurement of pornography attitudes and consumption patterns among youth in Malaysia. The outcomes from the study’s questionnaire can be used as a guide for establishing a more focused intervention plan for pornography prevention and control among youth in Malaysia.

## Methods

This study was conducted in Kelantan, a state located in the north-eastern part of Peninsular Malaysia and involved two phases: i) phase 1, which consisted of questionnaire development and ii) phase 2, which was comprised of validation studies, including exploratory and confirmatory analysis. According to the World Health Organization (WHO), ‘youth’ can be defined as a person aged between 15 and 24 years, while ‘adolescent’ can be defined as an individual between 10 and 19 years of age. ‘Pornography’ refers to any material that depicts sexual activity or erotic behaviour (3).

### Questionnaire Development

**Figure 1:**
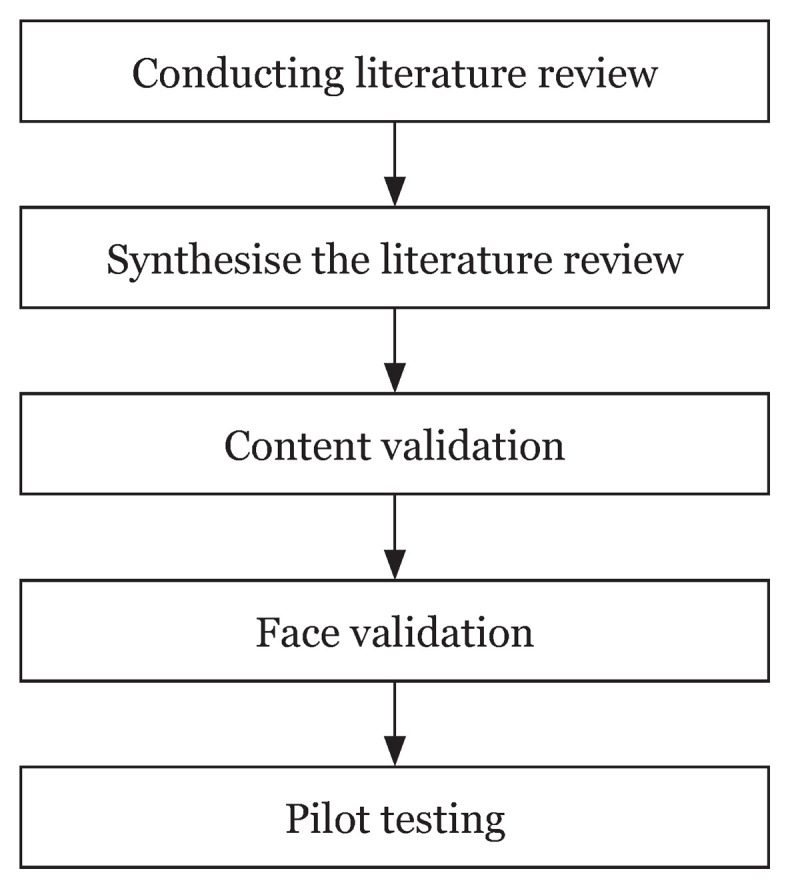
Questionnaire development using Amee guidelines

Phase 1 of the study was conducted between September 2017 and May 2018. The development of the questionnaire involved a few steps from the Amee guidelines (Figure 1). A thorough literature review on pornography related-topics, such as the prevalence of pornography, pornography attitudes, pornography patterns and factors related to pornography, were conducted. The literature search was carried out using Google Scholar, Science Direct, the National Library of Medicine (PubMed) and Scopus, which covered pornography topics at both local and global levels. Based on the literature review, the first draft of the questionnaire was developed.

The experts were i) two public health physicians; ii) two family medicine specialists with interest in sexual health; iii) a psychologist involved in school programmes and iv) a counsellor from a secondary school. The contents of the items were discussed according to the needs and understanding of the youth and took into consideration the suitability and sensitivity of local cultures and religions. The expert panels also examined the content validity of each item in the questionnaire in order to ensure their relevance, clarity, importance and completeness. Upon achieving consensus from the panel experts, the operational definition for pornography used in this study was set: ‘any material (e.g. in writing, photography or movies) that depicts sexual activity or erotic behaviour in a way that is designed to arouse sexual excitement’. ‘Pornography exposure’, on the other hand, refers to viewing or reading pornography at least once in a lifetime, be it intentional or unintentional.

The revised questionnaire on pornography (PORQUE), was set as a self-administered questionnaire, which follows the standard protocol for questionnaire design and testing. It encompassed items related to socio-demographic and family background, family relationship, religiosity, personal risky behaviours (e.g. high-risk behaviour and sexual risk behaviour), internet and phone usage, pornography consumption patterns and attitudes toward pornography. Details of the questionnaire are shown in Table 1.

After content validation, cognitive debriefing was conducted, whereby 10 youth were selected by convenient sampling for individual interviews. The purpose of the interviews was to test the face validity of each questionnaire item through an open-ended discussion. The participants were then asked to discuss and interpret each questionnaire item separately, and their responses and understanding of the items were evaluated.

A pilot test was carried out after the questionnaire was revised following face validation. There were 10 institutions identified in Kota Bharu, Kelantan. The pilot testing was conducted in a randomly selected higher learning institution and involved 50 respondents. During the pilot test, a few items were evaluated: i) technical aspects of the questionnaires (e.g. appropriateness of the wording used, the format of the questionnaires, the flow of the questions); ii) administration process (e.g. length and amount of time taken to complete the questionnaires and flow of questionnaire administration) and iii) data entry preparation (e.g. questionnaire coding, data entry procedure and frequency of non-response items).

### Validation Studies

#### Exploratory factor analysis (EFA)

Phase 2 of the study was conducted between June 2018 and September 2018. The first part of the validation study was conducted between June 2018 and July 2018 to explore the psychometric properties of the questionnaire. Another 150 youth were recruited from the same institution at which the pilot study was conducted. The inclusion criteria for the study was youth aged between 16 and 24 years old who consented to participate in the study, whereas the exclusion criteria included those who did not understand the Malay language, were not Malaysian or did not attend classes within the data collection period.

The participants who consented to participate were briefed on the study. Specifically, a few important terminologies related to the questionnaires, such as ‘pornography’, ‘intentional/unintentional exposure’ and ‘sex education’ were explained. The questionnaire was designed to be self-administered and anonymous. All participants were ensured of their confidentiality and that none of their details (e.g. name, identification or phone number) were recorded. This was done to increase the reliability of the responses.

All data obtained from each participant was entered using SPSS software (version 24, Armonk, NY IBM Corp, Statistical Package for the Social Sciences, USA). The data from Part B of the questionnaire (questions related to attitudes toward pornography) were then transferred to R version 3.5.1 (R Foundation for Statistical Computing, Vienna, Austria). Data exploration and cleaning were conducted to identify incorrect entries missing values and outliers. All negative items scores were reversed prior to analysis. The items in each attitude section were treated as continuous responses to allow for an evaluation of the dimensionality (number of factors) of the items. Descriptive analyses were conducted to identify for the minimum-maximum values per item as well as the frequency and percentage of response to the options per item.

To ensure the suitability of the data, Kaiser-Meyer-Olkin (KMO) and Bartlett’s test of sphericity were used (16). KMO values > 0.7 and a Bartlett’s test of sphericity with a *P*-value < 0.05 indicated the data was suitable for analysis. Meanwhile, to determine the number of extracted factors, Eigenvalues > 1.0, parallel analysis and screen plot inspection were performed (17).

The principal axis factoring (PAF) extraction method with Oblimin rotation was applied to extract the factors (18). This extraction method was used because it does not assume normally distributed data (17). The quality of the items was assessed based on factor loadings, communalities values and factor correlations. Factor loadings > 0.5, communalities > 0.25 and factor correlations < 0.85 were considered acceptable values (16–18). Items with good factor loadings and/or clinical importance were retained and vice versa. Repeat analysis was done whenever the removal of items occurred. For internal consistency reliability, a Cronbach’s alpha coefficient of > 0.7 was considered acceptable (19).

#### Confirmatory factor analysis (CFA)

In the second part of the validation study, the revised PORQUE was administered to another 198 respondents from the same higher learning institution. This study aimed to further explore and confirm the psychometric properties of the questionnaire.

Similarly, the respondents were briefed and consent was obtained from each participant. The attitude questionnaire was analysed by CFA using the Lavaan package version 0.6–3.0 (R Development Core Team, 2016). The model fit assessment was based on the following fit indices and their respective cut-off values: χ^2^ *P* > 0.05, a comparative fit index (CFI) and Tucker-Lewis fit index (TLI) close to or more than 0.95, a root mean square error of approximation (RMSEA) ≤ 0.08 and a standardised root mean square residual (SRMR) ≤ 0.08. Raykho’s rho was used for composite reliability (20) using the semTools package version 0.5–0 (R Development Core Team, 2016). A composite reliability value ≥ 0.7 was considered acceptable (20).

## Results

### Questionnaire Development: Content and Face Validity and Pilot Testing

A thorough and extensive literature search of the different subtopics under pornography was helpful in identifying important issues to be highlighted in the formation of constructs and item selection for the attitude questionnaire. Prior to content validation, a draft of the questionnaire was prepared to assist with further discussion during expert meetings. Therefore, during content validation, the panel of experts discussed and judged the initial questionnaire and unanimously agreed to add a few items to the questionnaire while maintaining the good content validity of each item according to their relevancy, clarity, importance and completeness.

During cognitive debriefing or face validation, a few ambiguous terms were highlighted by the respondents such as ‘sex education’ and ‘masturbation’. Thus, the questionnaire was improved by providing additional information on or a definition of such terms. During the pilot test, the duration of response to complete the questionnaire ranged from 10 min – 30 min with an average of 20 min. A few technical aspects, such as font size, spacing and questionnaire flow, were changed. For example, questions on pornography consumption patterns were changed to be located after the attitude questions, as they were more related to pornography practice. To avoid the frequency of non-response items, all items under the ‘attitude’ umbrella were divided into sub-domains to gain better feedback from the respondents. The coding for each questionnaire item was prepared prior to data entry. The details of such questionnaire changes are summarised in Table 2.

### Questionnaire Validation: EFA and CFA

The socio-demographic pattern of the respondents for both the EFA and CFA is shown in Table 3. The mean age for both EFA and CFA was 19.6 and 20.4, respectively. Both genders were represented in almost similar proportions in the EFA, whereas, in the CFA, male respondents comprised two-thirds of the total respondents. The respondents were all Muslims, the majority of which resided in Kelantan. In terms of parents’ education, the majority of the respondents had parents who had at least completed secondary school, which was consistent with the mean total household income of RM1,400–RM2,000 per month. Most of the respondents’ mothers worked as housewives, whereas their fathers represented several different job categories.

During analysis of the attitude questions, principle axis factoring (PAF) was used with the Oblimin method to test for multi-collinearity. The KMO test was 0.766 and the Bartlett’s test of sphericity was significant (*P*-value < 0.001). The EFA suggested a five-factor solution, whereas the screen-plot inspection suggested a six-factor solution. Thus, the factor numbers were fixed to five, as per our discussion from the experts meeting. Based on the tri-factor model of attitude, the first 20 items were grouped under the Affect factor (with two sub-domains), whereas another 36 items were grouped under the Cognitive factor (with three sub-domains). Thus, overall, there were two affect factors (e.g. permissive and non-permissive feelings toward pornography) and three cognitive factors (e.g. permissive perception of pornography, perception of impact of reading or watching pornography and perception of factors contributing to pornography). Based on the EFA, the factor loadings for each item were > 0.3 and the communalities were > 0.25. There were no items with cross loadings. The results in terms of the factor loadings, communalities and Cronbach’s alpha are shown in Table 4.

During CFA analysis, the z kurtosis in Mardia’s test was > 0.5 (62.67), which means the data did not exhibit multivariate normality. Thus, the robust maximum likelihood (MLR) estimation method was used for analysis. CFA analysis was done by using a five-factor model based on our results from the EFA. In order to obtain a good fit model, 17 pairs of correlated errors were added (as shown in Table 5). Most of the correlated errors took on similar yet different meanings in the sentences. For example, the word *membaca* or ‘reading’ was changed to *melihat* or ‘watching’ in the following sentences to emphasise the different meanings of the items. These similarities can be seen in correlated error numbers 1 to 13.

Similarities were also observed in other correlated errors (i.e. correlated error number 14: the word *meluangkan masa dengan anak-anak* or ‘spending time with children’ was changed to *rapat dengan anak-anak* or ‘close with children’; correlated error number 15: the word ‘internet’ was changed to *telefon bimbit* or ‘mobile phones’; correlated error number 16: the word *anak luar nikah* or ‘out of wedlock child’ was changed to *hamil luar nikah* or ‘premarital pregnancy’; correlated error number 17: the word *sex bebas* or ‘promiscuous sexual intercourse’ was changed to ‘pedophilia’.

**Figure 2:**
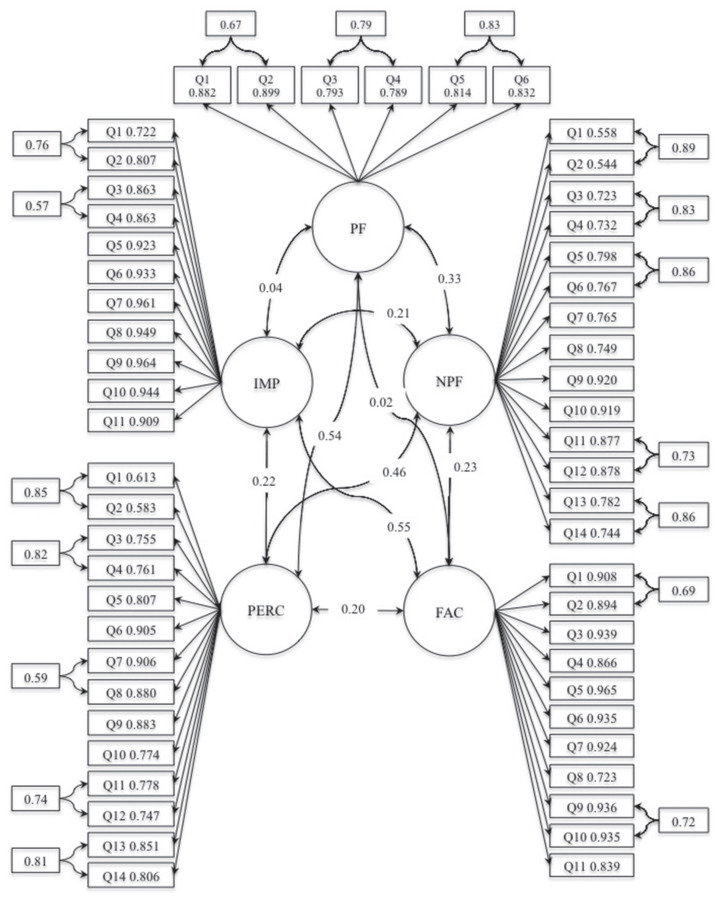
CFA diagram Notes: PERC = perception towards pornography; FAC = perception on factors contributed to pornography; IMP = perception on impact of pornography; NPF = non-permissive perception towards pornography; PF = permissive perception towards pornography

Following the addition of the correlated errors, as shown in Table 5, the model showed demonstrated good fit indices: *P*-value (Chi-square) < 0.001; CFI robust = 0.907; TLI robust = 0.901; RMSEA robust = 0.073; and SRMR = 0.060. The correlation between the Affect factor and Cognitive factor (with each sub-domain) ranged from 0.036 to 0.551. Details on the inter-factor correlations are explained in Figure 2. All factor loadings for the items were > 0.5 and the composite reliability of all factors was > 0.7 (Table 6).

The overall results for the CFA are illustrated in Figure 2.

## Discussion

The main objective of this study was to develop a new Malay version of PORQUE that addresses the attitudes, consumption patterns, and factors associated with pornography of youth. This questionnaire was different from those in previous studies in Malaysia, one of which used general questions on pornography consumption as part of health screening for adolescents (21), while another was a qualitative study that focused on sexual initiation among adolescents rather than pornography (22). Overall, we managed to complete two phases of this study with adequate sample and responses to produce a valid tool to assess the attitudes and consumption patterns related to pornography among youth.

The Malay version of the questionnaire consisted of seven segments: socio-demographic and family background, family relationship, religiosity, personal risky behaviour, internet and phone usage, pornography exposure, and consumption patterns and attitudes toward pornography. The socio-demographic pattern of the respondents during the study’s EFA showed an equal gender distribution, whereas, in the CFA, the male respondents comprised two-thirds of the total respondents. This could be explained by the fact that the participants were recruited via convenient sampling. The first part of the validation, EFA, exhibited a good psychometric property of the attitude questions: EFA suggested a five-factor solution (two affect domains and three cognitive domains) with a good range of factor loadings and Cronbach’s alpha. The second part of the validation of the attitude questions, CFA, also exhibited a good fit model with good fit indices. The factor loadings and Raykov’s rho also showed a good range.

During the development process, much emphasis was placed on the content validation of the experts, whereby generations of the items were discussed in greater depth in each section. For example, each item was checked for its relevancy, clarity, importance and adequacy. Thus, each sentence was designed to be brief, clear and simple enough for the youth to understand and provide an appropriate response (15). The word ‘pornography’ was changed to *bahan-bahan lucah* or ‘explicit/obscene materials’, as it this was thought to be more palatable in Malaysian culture. During the development of the items for the attitude questionnaire, we noted the possibility of having two questions for the same item (23). For example, items with similar content were split into two separate questions for *membaca* ‘reading’ or *menonton* ‘watching’.

Due to the sensitive nature of this study, the questionnaire was designed to enable the researchers’ ability to detect a ‘faking index’ (i.e. untruthful responses given by participants) to avoid social response bias (17, 24–25). For example, during the development of the questionnaire for pornography consumption patterns, questions related to pornography exposure, intentional or unintentional use and consumption patterns were provided continuously without provisional instructions. This approach was used to avoid prejudice among respondents and encourage them to answer the questions without hesitance.

Based on a thorough questionnaire validation, we found that the assessment of the psychometric properties in the questionnaire was good when applied to the attitude section. The analysis of the attitude questionnaire resulted in a good-fitting five-factor model based on the EFA and CFA, in which two factors were clustered under the Affect model and another three under the Cognitive model (26).

The five main domains included in the attitude section were ‘permissive feelings toward pornography’ (14 items), ‘non-permissive feelings toward pornography’ (6 items), ‘permissive perception of pornography’ (14 items), ‘perception of impact of pornography’ (11 items) and ‘perception of factors that contribute to pornography’ (11 items). The psychometric property results for the domains ‘permissive feelings toward pornography’ and ‘non-permissive feelings toward pornography’ were consistent with findings from previous observational studies (6–7, 12). However, we were unable to compare the other three domains, as no similar studies had been conducted to assess the psychometric properties of such items.

We identified a few limitations in this study: first, it used convenient sampling; thus, even though the confidentiality of the study was ensured, there was still the possibility of social desirability bias, as the respondents may have felt pressured to answer the questionnaire due to peer factors and thus did not respond to the questions accordingly. Secondly, as this study relied on self-reported measures to maintain the anonymity of the respondents, we were unable to verify the responses, which may have limited our interpretation of the data.

## Conclusion

The newly developed PORQUE is reliable in terms of its satisfactory EFA and CFA results and is thus valid for use among Malay youth in Malaysia. However, due to the homogeneity of our respondents, this study may need to be repeated to generalisability to other target groups and races.

## Figures and Tables

**Table 1:** The PORQUE after content validation

No	Sections	No of items	Concepts measured	Response options
1.	General information	19	Socio-demographic, family background, parent education background and total household income	Close ended, multiple choice
2.	Family relationship	2	Family closeness, to whom they share their problems	Multiple choice
		4	Perception on parents care, whether the parents know their friends	Yes/No/Unsure
3.	Religiosity	5	Praying five times a day, fasting during month of Ramadan and reading Quran for Muslims, practicing their religion for non-Muslim	Yes/No/Unsure
4.	High risk behaviour	3	Smoking, substance abuse, history of illegal race	Yes/No/Unsure
5.	Sexual risk behaviour	4	History of being molested/rape, sex fantasy, history of masturbation, history of sexual intercourse	Yes/No/Unsure
6.	Internet and mobile phone use	6	History of internet use, choice of internet use, common application used, duration of internet use, internet watch accompany, location of internet use	Close ended, multiple choice
7.	Pornography consumption pattern	13	Pornography exposure, intention of pornography exposure, age of first exposure, who introduce pornography, reason of watching/reading pornography, duration of pornography, last exposure to pornography, medium of pornography exposure, source of pornography, location of pornography exposure, who accompany, sharing information on pornography	Close ended, multiple choice
8.	Attitudes towards pornography	55	Attitudes towards pornography	1=strongly disagree, 2=disagree, 3=unsure, 4=agree, 5=strongly agree

**Table 2:** Summary of PORQUE according to steps in development process

Sections	Concepts measured	Response options	Lite review	Content validation	Face validation	Pilot test	Final draft for EFA

			Number of items and comments				
General information	Socio-demo-graphic, family background, parent education background and occupation, total household income	Close ended, multiple choice	13	19	19	Boxes and fonts were enlarged, spacing increased, example given for open ended questions	22
Family relationship	Family closeness, to whom they share their problems	Multiple choice	1	2	2	Option boxes were omitted	2
	Perception on parents care, love, and whether the parents know their friends	Yes/No/Unsure	2	4	4	Option boxes with numbers	4
Religiosity	Praying five times a day, fasting during month of Ramadan and reading Quran for Muslims, practising their religion for non Muslim	Yes/No/Unsure	3	5	5	Option boxes with numbers	5
Personal risky behaviour	Smoking, substance abuse, history of illegal race, history of being molested/rape, sex fantasy, history of masturbation, history of sexual intercourse	Yes/No/Unsure	7	7	7 (masturbation defined)	Molested and raped history in separate questions	8
Internet and mobile phone use	History of internet use, choice of internet use, common application used, duration of internet use, internet watch accompany, location of internet use	Close ended, multiple choice	2	5	5	Questions on location of internet use was omitted	4
Pornography consumption pattern	Pornography exposure, intention of pornography exposure, age of first exposure, who introduce pornography, reason of watching/reading pornography, duration of pornography, last exposure to pornography, medium of pornography exposure, source of pornography, location of pornography exposure, who accompany, sharing information on pornography	Close ended, multiple choice	12	12	12	12	12
Attitudes toward pornography	General attitudes towards pornography	1 = strongly disagree, 2 = disagree, 3 = unsure, 4 = agree, 5 = strongly agree	52	55 (6 items removed, 8 items added)	55 (sex education defined, double barrel questions removed, question were rearranged under separate domains)	4 domains (Domain 1 – 34 items; Domain 2– 14 items; Domain 3 – 11 items; Domain 4 – 11 items)	4 domains (Domain 1–20 items with 6 items reverse score; Domain 2 – 14 items reverse score; Domain 3 – 11 items; Domain 4–11 items)

**Table 3:** Socio-demographic characteristic of respondents in validation phase

Variables	EFA *n* = 150 *N* (%)	CFA *n* = 198 *N* (%)
Age (year)	19.6 (0.7)^a^	20.4 (1.6)^a^
Gender		
Male	74 (49.3)	131 (66.0)
Female	76 (50.7)	67 (34.0)
Race		
Malay	150 (100)	193 (97.5)
Non-Malay	0	5 (2.5)^b^
State		
Kelantan	138 (92.1)	150 (75.5)
Others	12 (7.9)	48 (24.5)
Religion		
Muslim	150 (100)	198 (100.0)
Mother’s education		
Never schooling	7 (4.7)	7 (3.7)
Primary school	23 (14.9)	8 (4.3)
Secondary school	99 (66.9)	132 (70.7)
Diploma/Degree	19 (12.2)	33 (17.6)
Master/PhD	2 (1.4)	7 (3.7)
Father’s education		
Never schooling	10 (6.8)	11 (5.9)
Primary school	16 (11.0)	7 (3.7)
Secondary school	94 (64.4)	119 (63.8)
Diploma/Degree	23 (15.8)	42 (22.3)
Master/PhD	3 (2.1)	8 (4.3)
Mother’s occupation		
Housewife	108 (71.5)	135 (69.2)
Government/Private	15 (9.9)	36 (18.5)
Business	14 (9.3)	11 (5.6)
Self-employed	8 (6.0)	9 (4.6)
Others	5 (3.3)	4 (2.1)
Father’s occupation		
Government/Private	31 (20.4)	71 (37.0)
Unemployed	11 (7.5)	8 (4.1)
Business	25 (16.3)	22 (11.5)
Self-employed	49 (32.7)	74 (38.5)
Others	34 (23.1)	17 (8.9)

Total household income (RM)	1,447.1 (1,391.4)^a^	2,026.1 (1,911.6)^a^

Notes:

a mean (standard deviation);

b Bumiputera Sabah

**Table 4:** Extracted factors, factor loadings, communalities and reliability (*n* = 150)

Factor	Item	Factor loading	Communality	Cronbach’s alpha
1. Non permissive feelings toward pornography	Q1: Fikiran saya terganggu selepas membaca bahan-bahan lucah *I feel disturbed after reading pornography materials*	0.554	0.413	0.966
	Q2: Fikiran saya terganggu selepas menonton bahan-bahan lucah *I feel disturbed after watching pornography materials*	0.542	0.370	
	Q3: Saya rasa malu selepas membaca bahan-bahan lucah *I feel shy after reading pornography materials*	0.793	0.627	
	Q4: Saya rasa malu selepas menonton bahan-bahan lucah *I feel shy after watching pornography materials*	0.791	0.609	
	Q5: Saya terkejut bila terbaca bahan-bahan lucah *I feel shock when accidentally read pornography materials*	0.746	0.581	
	Q6: Saya rasa berdebar bila menonton bahan-bahan lucah *I feel palpitation after watching pornography materials*	0.560	0.347	
	Q7: Saya rasa murung bila membaca bahan-bahan lucah *I feel depress after reading pornography materials*	0.671	0.446	
	Q8: Saya rasa murung bila menonton bahan-bahan lucah *I feel depress after watching pornography materials*	0.710	0.506	
	Q9: Saya rasa menyesal bila membaca bahan-bahan lucah *I feel guilty after reading pornography materials*	0.748	0.590	
	Q10: Saya rasa menyesal bila menonton bahan-bahan lucah *I feel guilty after watching pornography materials*	0.751	0.597	
	Q11: Saya rasa jijik apabila terbaca bahan-bahan lucah *I feel gross after accidentally read pornographic materials*	0.761	0.686	
	Q12: Saya rasa jijik apabila terlihat bahan-bahan lucah *I feel gross after accidentally read pornographic materials*	0.739	0.675	
	Q13: Saya rasa berdosa bila membaca bahan-bahan lucah *I feel sinful after reading pornographic materials*	0.702	0.519	
	Q14: Saya rasa berdosa bila menonton bahan-bahan lucah *I feel sinful after watching pornographic materials*	0.620	0.403	

2. Permissive feelings toward pornography	Q15: Saya rasa teruja bila membaca bahan-bahan lucah *I feel excited after reading pornographic materials*	0.661	0.618	0.929
	Q16: Saya rasa teruja bila menonton bahan-bahan lucah *I feel excited after watching pornographic materials*	0.658	0.512	
	Q17: Saya rasa ghairah bila membaca bahan-bahan lucah *I feel passionate after reading pornographic materials*	0.794	0.662	
	Q18: Saya rasa ghairah bila menonton bahan-bahan lucah *I feel passionate after watching pornographic materials*	0.731	0.674	
	Q19: Saya berasa ingin mencuba aksi seks selepas membaca bahan-bahan lucah *I feel like trying sexual acts after reading pornographic materials*	0.602	0.615	
	Q20: Saya berasa ingin mencuba aksi seks selepas menonton bahan-bahan lucah *I feel like trying sexual acts after watching pornographic materials*	0.611	0.620	

3. Permissive perception towards pornography	Q21: Membaca bahan-bahan lucah adalah tidak salah *Reading pornographic materials is not wrong*	0.596	0.360	0.924
	Q22: Menonton bahan-bahan lucah adalah tidak salah *Watching pornography is not wrong*	0.624	0.417	
	Q23: Membaca bahan-bahan lucah adalah penting bagi remaja seperti saya *Reading pornographic materials is important for a teenager like me*	0.795	0.564	
	Q24: Menonton bahan-bahan lucah adalah penting bagi remaja seperti saya *Watching pornographic materials is important for a teenager like me*	0.743	0.514	
	Q25: Saya lebih senang memahami kandungan daripada bahan-bahan lucah berbanding dengan pendidikan seks di sekolah *I can easily understand the contents from pornographic materials than sex education at schools*	0.647	0.533	
	Q26: Saya ingin tahu lebih banyak lagi maklumat mengenai seks dengan membaca bahan-bahan lucah *I wish to know more information on sex by reading pornographic materials*	0.692	0.672	
	Q27: Saya ingin tahu lebih banyak lagi maklumat mengenai seks dengan menonton bahan-bahan lucah *I wish to know more information on sex by watching pornographic materials*	0.754	0.727	
	Q28: Pengetahuan saya mengenai seks meningkat bila membaca bahan-bahan lucah *My knowledge on sex increase after reading pornographic materials*	0.606	0.541	
	Q29: Pengetahuan saya mengenai seks meningkat bila menonton bahan-bahan lucah *My knowledge on sex increase after watching pornographic materials*	0.595	0.538	
	Q30: Saya boleh menjana pendapatan dari aktiviti berkaitan penyebaran bahan-bahan lucah *I can generate income from activities related to pornography*	0.553	0.448	
	Q31: Saya rasa aktiviti membaca bahan-bahan lucah adalah lebih baik daripada berzina/melakukan hubungan seks *I feel that reading pornographic materials is better than comitting sexual act*	0.571	0.433	
	Q32: Saya rasa aktiviti menonton bahan-bahan lucah adalah lebih baik daripada berzina/melakukan hubungan seks *I feel that watching pornographic materials is better than comitting sexual act*	0.534	0.470	
	Q33: Membaca bahan-bahan lucah boleh menghilangkan stress *Reading pornographic materials can release stress*	0.513	0.594	
	Q34: Menonton bahan-bahan lucah boleh menghilangkan stress *Watching pornographic materials can release stress*	0.449	0.357	

4. Perception on impact of pornography	Q35: Bahan-bahan lucah adalah punca kepada anak luar nikah *Pornography is the cause of illegal child*	0.822	0.634	0.907
	Q36: Bahan-bahan lucah adalah punca kepada hamil luar nikah *Pornography is the cause of out-of-wedlock marriage*	0.891	0.730	
	Q37: Bahan-bahan lucah adalah punca kepada seks bebas *Pornography is the cause of free sex*	0.857	0.765	
	Q38: Bahan-bahan lucah adalah punca kepada *paedophilia* *Pornography is the cause of paedophilia*	0.879	0.746	
	Q39: Bahan-bahan lucah adalah punca kepada seks songsang (LGBT) *Pornography is the cause of LGBT*	0.838	0.721	
	Q40: Bahan-bahan lucah adalah punca kepada aktiviti seks sebelum nikah *Pornography is the cause of sex before marriage*	0.853	0.798	
	Q41: Bahan-bahan lucah adalah punca kepada kes rogol *Pornography is the cause of rape cases*	0.842	0.750	
	Q42: Bahan-bahan lucah adalah punca kepada ketagihan terhadap seks *Pornography is the cause of sex addiction*	0.863	0.720	
	Q43: Bahan-bahan lucah adalah punca kepada keganasan seksual *Pornography is the cause of sex violence*	0.813	0.762	
	Q44: Pendedahan kepada bahan lucah adalah punca lelaki, wanita dan kanak-anak dianggap sebagai objek seks *Exposure to pornography is the cause of women, men and children being regarded as sex objects*	0.805	0.729	
	Q45: Bahan-bahan lucah adalah adalah punca kepada eksploitasi lelaki/wanita/kanak-kanak untuk aktiviti seks *Pornography is the cause of sex exploitation in men/women/children*	0.837	0.792	

5. Perception on factors contributed to pornography	Q46: Pemantauan ibu bapa dalam aktiviti penggunaan internet dapat mengelakkan pendedahan kepada bahan lucah *Parents monitoring on internet use can prevent exposure to pornography*	0.598	0.590	0.904
	Q47: Pemantauan ibu bapa dalam aktiviti penggunaan telefon bimbit dapat mengelakkan pendedahan kepada bahan lucah *Parents monitoring on mobile phone use can prevent exposure to pornography*	0.578	0.584	
	Q48: Kurang didikan agama adalah punca kepada penggunaan bahan-bahan lucah *Lack of religious education is the cause of pornography use*	0.791	0.584	
	Q49: Kurang ikatan keluarga adalah punca kepada penggunaan bahan-bahan lucah *Lack of family ties is the cause of pornography use*	0.674	0.523	
	Q50: Kurang amalan agama adalah punca kepada penggunaan bahan-bahan lucah *Lack of religious practice is the cause of pornography use*	0.805	0.633	
	Q51: Kurang iman/nilai-nilai moral adalah punca kepada penggunaan bahan-bahan lucah *Lack of religiousity/moral values is the cause of pornography use*	0.768	0.624	
	Q52: Penggunaan internet berleluasa adalah penyebab tersebarnya bahan-bahan lucah *Extensive internet use is the cause of pornography widespread*	0.550	0.485	
	Q53: Pendidikan seks di sekolah dapat mengurangkan penggunaan bahan-bahan lucah *Sex education at schools can reduce the use of pornography materials*	0.491	0.233	
	Q54: Ibu bapa yang meluangkan masa dengan anak-anak dapat mengurangkan penggunaan bahan-bahan lucah di kalangan anak-anak *Parents who spend more time with their kids can reduce the use of pornography materials among their kids*	0.683	0.573	
	Q55: Ibu bapa yang rapat dengan anak-anak dapat mengurangkan penggunaan bahan-bahan lucah di kalangan anak-anak *Parents who are close with their kids can reduce the use of pornography materials among their kids*	0.682	0.594	
	Q56: Hubungan kekeluargaan yang tidak harmoni menyebabkan penggunaan bahan-bahan lucah di kalangan anak-anak *Family dysharmony is a cause for use of pornography materials among their kids*	0.658	0.526	

**Table 5:** Correlated error between items

No	Items with correlated error	Covariance
1	F1 - I feel disturbed after reading pornography materials	0.89
	F2 - I feel disturbed after watching pornography materials	
2	F3 - I feel shy after reading pornography materials	0.83
	F4 - I feel shy after watching pornography materials	
3	F7 - I feel depress after reading pornography materials	0.86
	F8 - I feel depress after watching pornography materials	
4	F11 - I feel gross after accidentally read pornographic materials	0.73
	F12 - I feel gross after accidentally watch pornographic materials	
5	F13 - I feel sinful after reading pornographic materials	0.86
	F14 - I feel sinful after watching pornographic materials	
6	F15 - I feel excited after reading pornographic materials	0.67
	F16 - I feel excited after watching pornographic materials	
7	F17 - I feel passionate after reading pornographic materials	0.79
	F18 - I feel passionate after watching pornographic materials	
8	F19 - I feel like trying sexual acts after reading pornographic materials	0.83
	F20 - I feel like trying sexual acts after watching pornographic materials	
9	P1 - Reading pornographic materials is not wrong	0.85
	P2 - Watching pornography is not wrong	
10	P3 - Reading pornographic materials is important for a teenager like me	0.82
	P4- Watching pornographic materials is important for a teenager like me	
11	P11 - I feel that reading pornographic materials is better than committing sexual act	0.74
	P12 - I feel that watching pornographic materials is better than committing sexual act	
12	P13 - Reading pornographic materials can release stress	0.81
	P14 - Watching pornographic materials can release stress	
13	P8 - My knowledge on sex increase after reading pornographic materials	0.59
	P9 - My knowledge on sex increase after watching pornographic materials	
14	RF9 - Parents who spend more time with their kids can reduce the use of pornography materials among their kids	0.72
	RF10 - Parents who are close with their kids can reduce the use of pornography materials among their kids	
15	RF1 - Parents monitoring on internet use can prevent exposure to pornography	0.69
	RF2 - Parents monitoring on mobile phone use can prevent exposure to pornography	
16	I1 - Pornography is the cause of illegal child	0.76
	I2 - Pornography is the cause of out-of-wedlock marriage	
17	I3 - Pornography is the cause of free sex	0.57
	I4 - Pornography is the cause of paedophilia	

**Table 6:** Factor loadings and reliability of the model in attitude questionnaire (*n* = 198)

Factor	Item	Factor loading	Raykov’s rho
1. Non permissive feelings toward pornography	Q1: Fikiran saya terganggu selepas membaca bahan-bahan lucah *I feel disturbed after reading pornography materials*	0.558	0.919
	Q2: Fikiran saya terganggu selepas menonton bahan-bahan lucah *I feel disturbed after watching pornography materials*	0.544	
	Q3: Saya rasa malu selepas membaca bahan-bahan lucah *I feel shy after reading pornography materials*	0.723	
	Q4: Saya rasa malu selepas menonton bahan-bahan lucah *I feel shy after watching pornography materials*	0.732	
	Q5: Saya terkejut bila terbaca bahan-bahan lucah *I feel shock when accidentally read pornography materials*	0.798	
	Q6: Saya rasa berdebar bila menonton bahan-bahan lucah *I feel palpitation after watching pornography materials*	0.767	
	Q7: Saya rasa murung bila membaca bahan-bahan lucah *I feel depress after reading pornography materials*	0.765	
	Q8: Saya rasa murung bila menonton bahan-bahan lucah *I feel depress after watching pornography materials*	0.749	
	Q9: Saya rasa menyesal bila membaca bahan-bahan lucah *I feel guilty after reading pornography materials*	0.920	
	Q10: Saya rasa menyesal bila menonton bahan-bahan lucah *I feel guilty after watching pornography materials*	0.919	
	Q11: Saya rasa jijik apabila terbaca bahan-bahan lucah *I feel gross after accidentally read pornographic materials*	0.877	
	Q12: Saya rasa jijik apabila terlihat bahan-bahan lucah *I feel gross after accidentally read pornographic materials*	0.878	
	Q13: Saya rasa berdosa bila membaca bahan-bahan lucah *I feel sinful after reading pornographic materials*	0.782	
	Q14: Saya rasa berdosa bila menonton bahan-bahan lucah *I feel sinful after watching pornographic materials*	0.744	

2. Permissive feelings toward pornography	Q15: Saya rasa teruja bila membaca bahan-bahan lucah *I feel excited after reading pornographic materials*	0.882	0.884
	Q16: Saya rasa teruja bila menonton bahan-bahan lucah *I feel excited after watching pornographic materials*	0.899	
	Q17: Saya rasa ghairah bila membaca bahan-bahan lucah *I feel passionate after reading pornographic materials*	0.793	
	Q18: Saya rasa ghairah bila menonton bahan-bahan lucah *I feel passionate after watching pornographic materials*	0.789	
	Q19: Saya berasa ingin mencuba aksi seks selepas membaca bahan-bahan lucah *I feel like trying sexual acts after reading pornographic materials*	0.814	
	Q20: Saya berasa ingin mencuba aksi seks selepas menonton bahan-bahan lucah *I feel like trying sexual acts after watching pornographic materials*	0.832	

3. Permissive perception towards pornography	Q21: Membaca bahan-bahan lucah adalah tidak salah *Reading pornographic materials is not wrong*	0.613	0.935
	Q22: Menonton bahan-bahan lucah adalah tidak salah *Watching pornography is not wrong*	0.583	
	Q23: Membaca bahan-bahan lucah adalah penting bagi remaja seperti saya *Reading pornographic materials is important for a teenager like me*	0.755	
	Q24: Menonton bahan-bahan lucah adalah penting bagi remaja seperti saya *Watching pornographic materials is important for a teenager like me*	0.761	
	Q25: Saya lebih senang memahami kandungan daripada bahan-bahan lucah berbanding dengan pendidikan seks di sekolah *I can easily understand the contents from pornographic materials than sex education at schools*	0.807	
	Q26: Saya ingin tahu lebih banyak lagi maklumat mengenai seks dengan membaca bahan-bahan lucah *I wish to know more information on sex by reading pornographic materials*	0.905	
	Q27: Saya ingin tahu lebih banyak lagi maklumat mengenai seks dengan menonton bahan-bahan lucah *I wish to know more information on sex by watching pornographic materials*	0.906	
	Q28: Pengetahuan saya mengenai seks meningkat bila membaca bahan-bahan lucah *My knowledge on sex increase after reading pornographic materials*	0.880	
	Q29: Pengetahuan saya mengenai seks meningkat bila menonton bahan-bahan lucah *My knowledge on sex increase after watching pornographic materials*	0.883	
	Q30: Saya boleh menjana pendapatan dari aktiviti berkaitan penyebaran bahan-bahan lucah *I can generate income from activities related to pornography*	0.774	
	Q31: Saya rasa aktiviti membaca bahan-bahan lucah adalah lebih baik daripada berzina/melakukan hubungan seks *I feel that reading pornographic materials is better than comitting sexual act*	0.778	
	Q32: Saya rasa aktiviti menonton bahan-bahan lucah adalah lebih baik daripada berzina/melakukan hubungan seks *I feel that watching pornographic materials is better than comitting sexual act*	0.747	
	Q33: Membaca bahan-bahan lucah boleh menghilangkan stress *Reading pornographic materials can release stress*	0.851	
	Q34: Menonton bahan-bahan lucah boleh menghilangkan stress *Watching pornographic materials can release stress*	0.806	

4. Perception on impact of pornography	Q35: Bahan-bahan lucah adalah punca kepada anak luar nikah *Pornography is the cause of illegal child*	0.722	0.967
	Q36: Bahan-bahan lucah adalah punca kepada hamil luar nikah *Pornography is the cause of out-of-wedlock marriage*	0.805	
	Q37: Bahan-bahan lucah adalah punca kepada seks bebas *Pornography is the cause of free sex*	0.863	
	Q38: Bahan-bahan lucah adalah punca kepada *paedophilia* *Pornography is the cause of paedophilia*	0.864	
	Q39: Bahan-bahan lucah adalah punca kepada seks songsang (LGBT) *Pornography is the cause of LGBT*	0.923	
	Q40: Bahan-bahan lucah adalah punca kepada aktiviti seks sebelum nikah *Pornography is the cause of sex before marriage*	0.932	
	Q41: Bahan-bahan lucah adalah punca kepada kes rogol *Pornography is the cause of rape cases*	0.961	
	Q42: Bahan-bahan lucah adalah punca kepada ketagihan terhadap seks *Pornography is the cause of sex addiction*	0.949	
	Q43: Bahan-bahan lucah adalah punca kepada keganasan seksual *Pornography is the cause of sex violence*	0.964	
	Q44: Pendedahan kepada bahan lucah adalah punca lelaki, wanita dan kanak-anak dianggap sebagai objek seks *Exposure to pornography is the cause of women, men and children being regarded as sex objects*	0.944	
	Q45: Bahan-bahan lucah adalah adalah punca kepada eksploitasi lelaki/wanita/kanak-kanak untuk aktiviti seks *Pornography is the cause of sex exploitation in men/ women/children*	0.909	

5. Perception on factors contributed to pornography	Q46: Pemantauan ibu bapa dalam aktiviti penggunaan internet dapat mengelakkan pendedahan kepada bahan lucah *Parents monitoring on internet use can prevent exposure to pornography*	0.901	0.972
	Q47: Pemantauan ibu bapa dalam aktiviti penggunaan telefon bimbit dapat mengelakkan pendedahan kepada bahan lucah *Parents monitoring on mobile phone use can prevent exposure to pornography*	0.892	
	Q48: Kurang didikan agama adalah punca kepada penggunaan bahan-bahan lucah *Lack of religious education is the cause of pornography use*	0.939	
	Q49: Kurang ikatan keluarga adalah punca kepada penggunaan bahan-bahan lucah *Lack of family ties is the cause of pornography use*	0.856	
	Q50: Kurang amalan agama adalah punca kepada penggunaan bahan-bahan lucah *Lack of religious practice is the cause of pornography use*	0.965	
	Q51: Kurang iman/nilai-nilai moral adalah punca kepada penggunaan bahan-bahan lucah *Lack of religiousity/moral values is the cause of pornography use*	0.935	
	Q52: Penggunaan internet berleluasa adalah penyebab tersebarnya bahan-bahan lucah *Extensive internet use is the cause of pornography widespread*	0.924	
	Q53: Pendidikan seks di sekolah dapat mengurangkan penggunaan bahan-bahan lucah *Sex education at schools can reduce the use of pornography materials*	0.723	
	Q54: Ibu bapa yang meluangkan masa dengan anak-anak dapat mengurangkan penggunaan bahan-bahan lucah di kalangan anak-anak *Parents who spend more time with their kids can reduce the use of pornography materials among their kids*	0.936	
	Q55: Ibu bapa yang rapat dengan anak-anak dapat mengurangkan penggunaan bahan-bahan lucah di kalangan anak-anak *Parents who are close with their kids can reduce the use of pornography materials among their kids*	0.935	
	Q56: Hubungan kekeluargaan yang tidak harmoni menyebabkan penggunaan bahan-bahan lucah di kalangan anak-anak *Family dysharmony is a cause for use of pornography materials among their kids*	0.839	
